# Concurrent Hematogenous Osteomyelitis, Deep Vein Thrombosis, and Septic Pulmonary Embolism in an Adolescent Male: A Rare Presentation

**DOI:** 10.7759/cureus.80057

**Published:** 2025-03-04

**Authors:** Ia Khurtsilava, Darejan Kanjaradze, Natia Tsirdava, Tistsino Parulava, Irakli Darsania, Ekaterine Gozalishvili

**Affiliations:** 1 Pediatrics, Tbilisi Pediatric Private Clinic, Tbilisi, GEO; 2 Pediatrics, Tbilisi Medical Academy, Tbilisi, GEO; 3 Pediatric Intensive Care Unit, Tbilisi Pediatric Private Clinic, Tbilisi, GEO; 4 Pediatrics/Pediatric Gastroenterology, Tbilisi Medical Academy, Tbilisi, GEO; 5 Pediatric Surgery, Tbilisi Pediatric Private Clinic, Tbilisi, GEO; 6 Pediatric Emergency Department, Pineo Medical Ecosystem, Tbilisi, GEO

**Keywords:** acute hematogenous osteomyelitis, deep vein thrombosis (dvt), pediatric case, pediatric intensive care unit(picu), septic pulmonary embolism

## Abstract

This case report describes a rare presentation of simultaneous hematogenous osteomyelitis, deep vein thrombosis (DVT), and septic pulmonary embolism in a 13-year-old male adolescent. While pediatric osteomyelitis is relatively common, the combination of venous thrombosis and pulmonary embolism is infrequently reported. Despite receiving antibacterial and antithrombotic treatment, the patient did not show any significant improvement. An MRI led to the diagnosis of osteomyelitis, and surgical intervention was performed. Post-surgery, the patient's clinical condition improved, enabling the removal of artificial ventilation and discharge from the clinic. The patient successfully returned to normal life with no neurological changes. This report highlights the need for timely diagnostic interventions and a comprehensive and collaborative approach to managing rare pediatric conditions. Raising awareness about such rare combinations is important to reduce the risk of complications in pediatric patients.

## Introduction

Osteomyelitis is a relatively common infectious disease seen in pediatric patients, whereas venous thrombosis is less prevalent in children compared to adults [1]. Pediatric venous thrombosis is often associated with triggering factors such as previous central venous catheter usage, trauma, hypercoagulable states, prolonged immobility, and chronic inflammatory conditions [1,2]. The occurrence of deep vein thrombosis (DVT) and septic pulmonary embolism concurrently with osteomyelitis is rarely observed in children, making it difficult to diagnose [3,4,5]. We describe a case of an adolescent male who initially presented with leg pain and a limp. Unlike many self-limiting cases, our patient had a very severe manifestation of the disease, which presented a diagnostic challenge for the clinicians.

## Case presentation

A 13-year-old male arrived at the emergency department by ambulance with a three-day history of high-grade fever, worsening left leg pain unresponsive to painkillers, and difficulty walking. He was residing in a rural area and engaged in activities such as hiking and wrestling. The patient's mother recalled that nearly two weeks ago, some skin on his knee had been damaged during a hiking trip, though the patient denied any recent serious trauma. Upon arrival at our emergency department, the patient had a temperature of 40.5 °C, tachycardia (200 beats per minute), weak peripheral pulses, prolonged capillary refill time (six seconds), and a respiratory rate of 40 breaths per minute. The patient had difficulty breathing, and oxygen saturation was 86% at room temperature. The left lower extremity was edematous, tender to palpation, and hyperemic.

Due to the severity of the condition, the patient was transferred to the pediatric ICU. Vesicular breath sounds were noted during the chest examination, with no crackling or wheezing. Additionally, a polymorphous rash was observed on the torso and extremities. Noninvasive ventilation with 10 PEEP (positive end-expiratory pressure) and intravenous rehydration were initiated. A cardiac examination revealed no structural abnormalities; however, sinus tachycardia ranging from 150 to 170 beats per minute was observed. An abdominal ultrasound revealed a slight increase in the size of both the liver and the spleen. The X-ray of the affected limb showed no abnormalities, while the Doppler examination of the lower extremity revealed thrombophlebitis, a diagnosis documented by the angiologist. Following initial laboratory tests, a course of antithrombotic and antimicrobial therapy was introduced: vancomycin 15 mg/kg intravenously (IV) every six hours plus cefepime 50 mg/kg IV every eight hours and enoxaparin) The patient's respiratory distress worsened over the next few hours, accompanied by a change in mental status. Following the administration of relaxants and sedation, invasive ventilation was initiated using pressure-support synchronized intermittent mandatory ventilation (SIMV-PSV).

**Figure 1:**
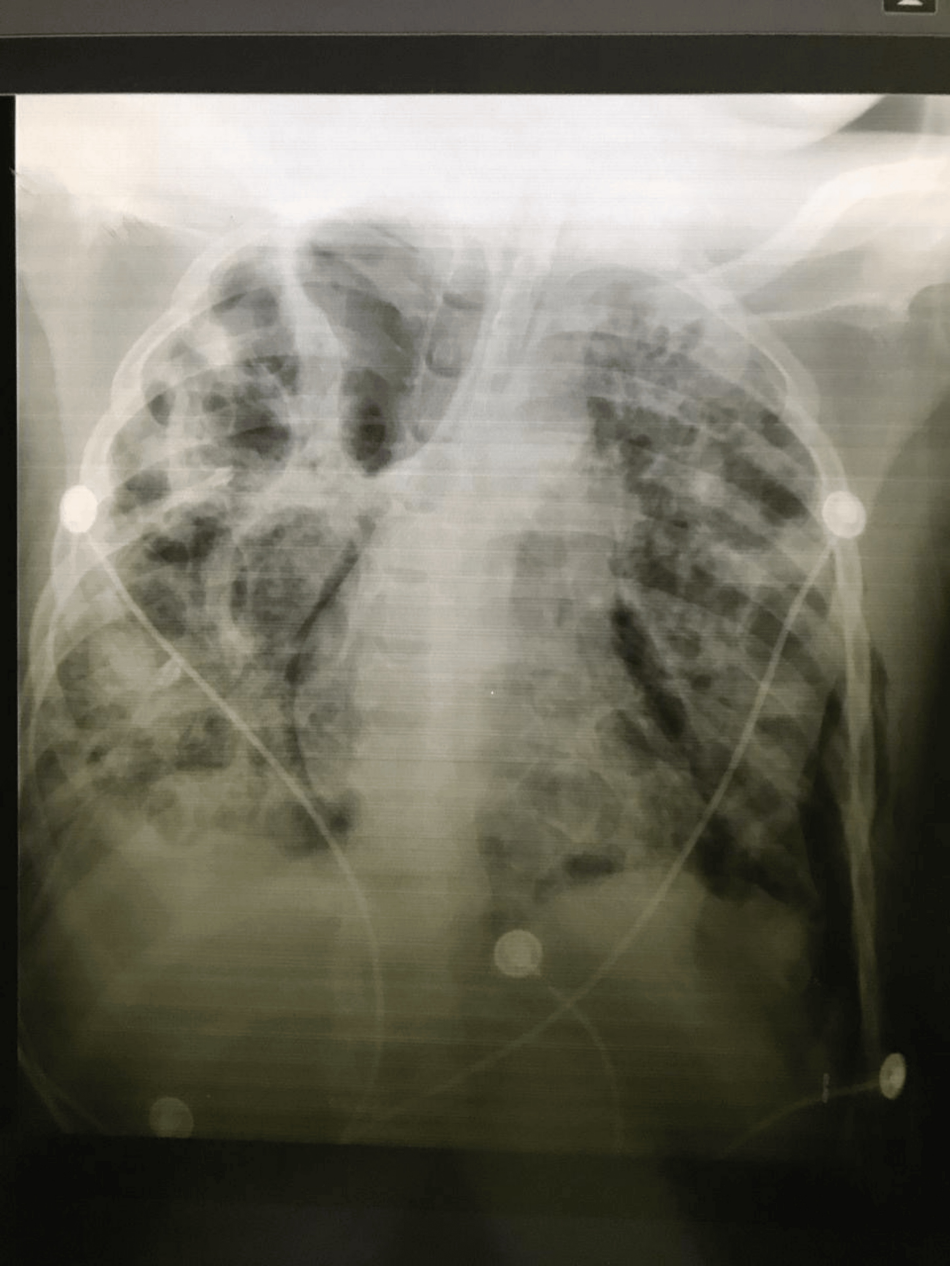
Initial chest X-ray

Initial laboratory tests revealed the following results (Table 1): urine analysis showed a light brown color, SG: 1.025, pH: 6.0, urobilinogen +++, bilirubin ++, ketones -, protein - 0.033%, nitrites -, glucose -, RBC: 1-2, and WBC 2-4. Additional results were as follows: anti-HIV1/2 (rapid test): negative, PT: 15.5 sec (11.8-18.6), PI: 84% (70-110), INR: 1.2 (0.87-1.43), APTT: 24.1 sec (<40), fibrinogen: 451 mg/dl (200-400), D-dimer: >10 (<0.5), ferritin: 1460.5 ng/ml (20-40), procalcitonin: 13.22 ng/ml (<0.5), total protein: 51.9 g/L (60-90), albumin: 20.7 g/L (32-45), ALT: 30.6 (<41 u/L), AST: 147.6 (<37 U/L), and total bilirubin: 35.8 (<187 mcmol/L). Lupus anticoagulants (LA) were 0.81 (<1.2). Serology results indicated anti-SARS-CoV-2 IgM-negative, anti-SARS-CoV-2 IgG-positive, VZV IgM-negative, and VZV IgG-negative. Blood cultures were sterile; sputum culture and bronchoalveolar lavage fluid showed no bacterial growth. Four days later, a chest X-ray (Figure 1) revealed tension pneumothorax, and intercostal drainage was obtained. Due to the patient's unstable condition, the planned CT scan was postponed. After a 10-day interval, a chest CT scan documented bilateral, diffuse pulmonary infiltrates, and the presence of abscesses.

**Table 1:** Patient's lab results on admission and after two months of treatment CRP: C-reactive protein; ESR: erythrocyte sedimentation rate; LDH: lactate dehydrogenase; MCV: mean corpuscular volume

Variable	On admission	Two months later	Reference range
Hemoglobin	135	127	12.4-16.4 g/L
Hematocrit%	33	49	40-51%
MCV	81	84	80-96 fL
Red cell count	4.4	4.5	4.00-5.50 10^12^/L
White cell count	24.6	7.5	5.0-10.0 10^9^/L
Neutrophils	97	42.8	50.0-70.0%
Lymphocytes	17	44.6	20-40%
Platelet count	153	283	150-400 10^9^/L
CRP	148.1	5.6	<6 mg/dl
ESR	20	12	<20 mm/hr
Creatinine	67	44	<88 µmol/L
Urea	13.3	3.2	2.76-8.07 mmol/L
Sodium	140	143	135-145 mmol/L
Potassium	3.6	3.9	3.4-5.00 mmol/L
D-dimer	>10	-	<5 μ/mL
Ferritin	1460.5	54	20-400 µg/L
LDH	910	199	120-300 IU/L

Despite multiple negative culture results, suspicion of a staphylococcal infection persisted. The patient's clinical condition failed to improve, even with antithrombotic and antimicrobial treatment. Draining was deemed necessary for the tension pneumothorax. A tracheostomy was carried out due to the requirement for prolonged artificial ventilation. MRI of the affected limb was scheduled. Our clinic does not have access to MRI scans, and transporting the patient for the required MRI was challenging due to the severity of the condition; hence, it was postponed until day 22 of the disease course. The MRI eventually led to the conclusive diagnosis of hematogenous osteomyelitis of the tibia. After multiple consultations with orthopedic surgeons, surgery was planned, which was performed two months after the onset of the initial course of the disease. Material from the proximal end of the left tibia was obtained during surgery, revealing focal necrotic destructive and infiltrating inflammatory changes along with extensive hemorrhages. The diagnosis of acute purulent osteomyelitis was established.

**Figure 2:**
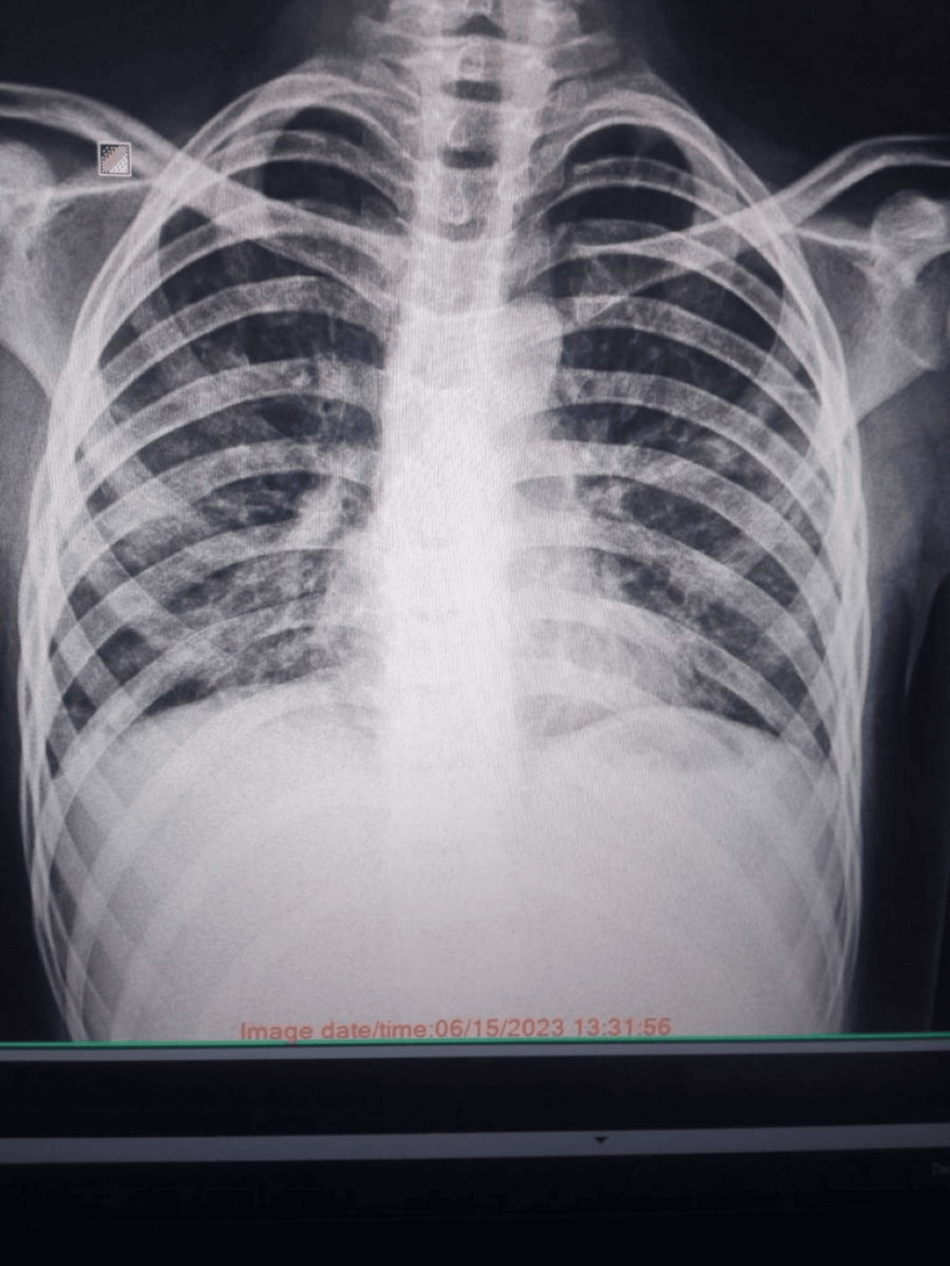
Chest X-ray on a follow-up visit

Postoperatively, the patient's condition remained stable. Subsequently, there was a notable clinical improvement, leading to the discontinuation of artificial ventilation and the removal of pleural drainages. Two weeks later, the patient was no longer dependent on oxygen. Initial antimicrobial therapy was discontinued. The tracheostomy tube was removed. The patient underwent continued monitoring for an additional two weeks and was discharged from the clinic. Levofloxacin and Fraxiparine were recommended for an additional two weeks. We are continuing to monitor the patient. He is leading a normal life and has successfully returned to school and actively participated in various school activities, though with some limitations. No neurological changes have been observed so far. Figure 2 shows the chest X-ray of the patient on a follow-up visit.

## Discussion

The presented case involves a rare and challenging scenario of a 13-year-old male with a simultaneous occurrence of osteomyelitis, DVT, and pulmonary complications. Pediatric osteomyelitis is relatively common, but its co-occurrence with venous thrombosis and pulmonary embolism in children is infrequently reported and is a diagnostic challenge for clinicians [4,5]. Admission to ICU, sepsis, higher D-dimer, higher body temperature during hospitalization, and methicillin-resistant Staphylococcus aureus (MRSA) are considered risk factors for thrombosis, and MRSA is an independent risk factor for DVT. For patients with related risk factors, timely ultrasound examination of the infected site should be considered for early detection and prompt treatment [6]. While this combination is rare, especially in the pediatric population, similar cases have been reported by the authors. In the vast majority of those cases, MRSA was isolated in cultures [7].

Despite multiple attempts, we were unable to isolate any microorganisms. The patient's initial presentation to the emergency department with high-grade fever, leg pain, and respiratory distress, along with tachycardia, weak peripheral pulses, prolonged capillary refill time, and low oxygen saturation, as well as an edematous and hyperemic left lower extremity and polymorphous rash, made reaching a diagnosis challenging for the team. Acute osteomyelitis complicated with DVT was suggested, as similar manifestations have been mentioned in multiple case reports [7]. Thrombophlebitis was diagnosed through a Doppler examination, and antithrombotic and antimicrobial therapy was subsequently initiated. Invasive ventilation was required due to the progression of the patient’s respiratory distress. Laboratory changes indicated a severe inflammatory response. Despite multiple negative culture results, suspicion of a staphylococcal infection remained, as it is considered the main cause according to multiple sources [6,7].

Antimicrobial therapy was initiated to cover possible causative organisms, but the patient's clinical condition did not improve; the MRI test required transport to another facility and was delayed due to the severity of the patient's condition. Subsequent findings of hematogenous osteomyelitis underscored the need to confirm diagnosis with an MRI scan. Surgery was performed two months after the initial diagnosis, and it revealed acute purulent osteomyelitis with necrotic destructive changes.

This case was a diagnostic challenge for the team, as it did not present the classical manifestation of acute osteomyelitis in the pediatric population, and these simultaneous complications are also rare. The inability to detect the causative organism also made it difficult to make a conclusive diagnosis. Following surgery, the patient's condition was stable and showed rapid clinical improvement, which in turn allowed the discontinuation of artificial ventilation, removal of pleural drainages, and discharge from the clinic. The timing of surgical intervention was challenging for the clinicians due to the severity of the patient’s condition, but notable clinical improvement raises concerns related to early surgical intervention in severe hematogenous osteomyelitis accompanied by thrombosis.

## Conclusions

A comprehensive and collaborative approach is required to manage rare and challenging pediatric conditions. The successful outcome in this case was achieved through timely recognition of the diverse clinical manifestations, appropriate diagnostic investigations, and adequate interventions. Raising awareness about such conditions is vital to ensure early diagnosis and management, which in turn can decrease the risk of complications, including sepsis, bone destruction, and the potential need for joint replacement surgery in the later stages of the disease.
